# Brain Size, Metabolism, and Social Evolution

**DOI:** 10.3389/fphys.2021.612865

**Published:** 2021-02-23

**Authors:** Zach N. Coto, James F. A. Traniello

**Affiliations:** ^1^Department of Biology, Boston University, Boston, MA, United States; ^2^Graduate Program in Neuroscience, Boston University, Boston, MA, United States

**Keywords:** brain metabolism, metabolic scaling, cognitive demands, social brain, ants

Questions concerning the integration and scaling relationships among size, morphology, physiology, and behavior are fundamental in evolutionary biology. The evolution of brain size and structure and their association with behavior is typically examined through analyses of encephalization, brain mosaicism, and allometries among functionally specialized brain compartments. Research across diverse clades has not clarified how brain size may evolve in response to environmental, social, and cognitive requirements. Studies of metabolic and neuroarchitectural scaling across multiple levels of organization can advance our understanding of the evolution of brain and behavior. However, the analysis of brain evolution has been limited by a lack of data on brain metabolism and its relation to brain and body size, and behavior. Using ants as eusocial insect exemplars, we propose an analysis of brain evolution encompassing metabolism, brain size, and neuroarchitecture to understand how these traits scale with body size and correlate with social behavior.

## Brain Size, Mosaicism, and Social Behavior

Variation in brain size and structure is correlated with ecology (Liao et al., 2015; Hoops et al., 2017; DeCasien and Higham, 2019) and sociality (Kolb et al., 2013; Kotrschal et al., 2014; Dunbar and Shultz, 2017). Significant associations among brain size, structure and social behavior have been found in insects (Ott and Rogers, 2010; Eberhard and Wcislo, 2011; Muscedere and Traniello, 2012; Feinerman and Traniello, 2015; Gordon et al., 2017). Limitations of volumetric correlations have been noted (Healy and Rowe, 2007; Logan et al., 2018). Studies of the structure and number of cells, synapses, and circuits may provide important neuroethological detail (Godfrey and Gronenberg, 2019), although comparative studies of individual neurons (Giraldo et al., 2013) and synaptic processing capability (Falibene et al., 2015; Gordon and Traniello, 2018; Gordon et al., 2019; Groh and Rössler, 2020) vary in degree of linkage with behavior. Volume (or mass) data are indeed required to understand scaling of cellular metrics such as synaptic density (Yilmaz et al., 2016) or neuromodulator titer, and can provide insight into sex-specific brain differentiation (Kiesow et al., 2020) and regional investment associated with social network size (Noonan et al., 2018). Metrics of brain size and mosaicism will continue to contribute to understanding brain architecture in sociobiological contexts.

Ranging in size from microscopic wasps to goliath beetles, insects provide diverse models for studying relationships among brain size, metabolism, and behavior (Rittschof et al., 2015; Rittschof and Schirmeier, 2017). Ants exhibit robust behavioral performance at miniature size and remarkable cognition as individuals (Giurfa, 2019) and groups (Sasaki and Pratt, 2018). Colonies may be composed of polymorphic workers that divide labor according to size and/or exhibit complex collective behaviors. The impact of worker cognitive demands and colony-level capabilities on brain size and metabolism has only begun to be investigated. Variation in ant worker size—from minute *Carebara atoma* to “huge” *Dinoponera*—and colony complexity provides outstanding opportunities to examine brain metabolism in relation to individual and group behavior.

Clade-specific brain scaling relationships, and their relation to behavior, require explanation. For example, diphasic allometry of brain mass to body mass indicates a significant increase in slope below a particular body size in some species; this may reflect limitations of investment in neural tissue in small bodies (Seid et al., 2011; van der Woude et al., 2013; Groothius and Schmid, 2017; Polilov and Makarova, 2017). Fungus-growing ants, whose workers may have task specializations associated with their agrarian habits, show a reduced allometric slope of brain mass to body mass relative to other ants and a higher body mass at which diphasic allometry occurs (Seid et al., 2011). Relative antennal lobe volume is positively correlated with colony size, but decreases in species with strong worker polymorphism and task specialization (Riveros et al., 2012). In the desert ant *Cataglyphis*, species forming large colonies have workers with significantly larger brains than those with small colonies (Wehner et al., 2007). These studies suggest that brain size, body size, behavior, colony size, and social complexity are related.

## Metabolic Scaling and Brain Operation Costs

Metabolism is considered highly significant to brain evolution because brains are energetically expensive (Aiello and Wheeler, 1995; Isler and van Schaik, 2009). Larger brains may allow increased information processing; therefore, the increased cost of larger brain size implies selection for cognition in vertebrates (Pontzer et al., 2016; Dunbar and Shultz, 2017; DeCasien and Higham, 2019) and insects (Niven and Laughlin, 2008). However, there are few studies on brain metabolic scaling (West et al., 2002; Prothero, 2015) that relate brain size and metabolism. Furthermore, it is unclear whether theories of brain metabolic scaling apply to insects, whose brain structure, neuron physiology, and small size may differ in information-processing ability from vertebrates (Laughlin et al., 1998; Faisal et al., 2005; Niven and Farris, 2012; Sengupta et al., 2013). Analyses of size-related constraints on metabolism (Darveau et al., 2002; West and Brown, 2005; Fonseca-Azevedo and Herculano-Houzel, 2012), life-history traits (Harrison, 2017), and brain size evolution (Harrison et al., 2002; Isler and van Schaik, 2006, 2009; Niven and Laughlin, 2008; Navarrete et al., 2011) will benefit from the integrative study of brain size, neuron structure and brain metabolism in insects.

Brain metabolism may relate to social complexity. Kamhi et al. (2016) used cytochrome oxidase (COX) activity, a proxy for neuron metabolism (Déglise et al., 2003), to contrast brain evolution and social evolution in the weaver ant *Oecophylla smaragdina* and the garden ant *Formica subsericea*, two sister clades whose workers are equivalent in body size but differ strongly in social organization and collective intelligence. Increased social complexity in weaver ants—reflected in division of labor by worker physical castes, large colony size, and remarkable group action—was associated with larger mushroom bodies (centers of higher-order information processing) exhibiting reduced COX activity. Increased brain size in a socially complex ant may thus be associated with reduced metabolic cost, contrasting with the assumption that increased brain size increases metabolic costs (Isler and van Schaik, 2009). Data on brain size and metabolism are therefore required to understand brain evolution.

## Brain Metabolism, Synaptic Plasticity, and Division of Labor

Subcaste-specific metabolic scaling patterns that correlate with division of labor (Shik, 2010) and worker age can offer insight into metabolic costs of behavior and synaptic structure. Minor workers of the dimorphic ant *Pheidole dentata* show an increase in the size and number of synapses and vesicles of individual presynaptic boutons without synaptic number change in the mushroom body lip over the first 3 weeks of adult life (Seid et al., 2005) when their task diversity (Seid and Traniello, 2006) and efficiency (Muscedere et al., 2009) increase. In contrast, *Pheidole* major workers exhibit a limited behavioral repertoire even at maturity (Mertl and Traniello, 2009). In both subcastes, age is associated with increased brain volume (Muscedere and Traniello, 2012) and declines in the density of mushroom body lip microglomeruli (Gordon et al., 2018)—synaptic structures that underlie plasticity in sensory processing capability and behavior (Groh and Rössler, 2011, 2020; Groh et al., 2014; Rössler, 2019). These changes are greater in minor workers. Synaptic transmission and plasticity are metabolically costly (Attwell and Laughlin, 2001; Harris et al., 2012; Todorova and Blokland, 2017). Therefore, increased brain metabolic rate during maturity due to increased brain size may be more pronounced in minors in association with synaptic changes underpinning their greater task diversity. Additionally, brain serotonergic immunoreactivity (Seid et al., 2008) and serotonin titers (Seid and Traniello, 2005; Muscedere et al., 2012) increase with age. Brain metabolic rate may decrease in response to increased serotonin activity (New et al., 2004; Rittschof et al., 2019) and decline with age in *P. dentata*. Recording brain and mushroom body metabolism in young and old minor and major workers can provide data to enhance our understanding of how synaptic energy costs relate to variation in neuropil volume, synaptic remodeling, and behavioral pluripotency.

## Future Research

Technical advances have increased the accuracy and sensitivity of metabolic research. Neville et al. (2017) recorded metabolism in intact *Drosophila* larval brains similar in volume (Cardona et al., 2010) to those of minute ant brains or large mushroom bodies (Arganda et al., 2020). Pooled samples may be needed for small compartments or, for example, to measure dissociated mitochondria activity (Rittschof et al., 2019). Additional methods can be applied. Barros et al. (2018) describe the use of 2-deoxyglucose and transgenic production of fluorescent metabolites to study brain metabolite fluxes, and at the organismal level, whole-body respirometry (Waters et al., 2017; Lighton, 2019) enables measurement of the metabolic rates of workers and colonies. Computational neuroimaging (Arganda-Carreras et al., 2017) accelerates collateral volumetric data collection for scaling studies. Proof of concept is clear, and a strong methodological foundation supports empirical studies on brain metabolism, size, and structure in ants. Variation in social complexity, worker body size, species richness, and ecology, as well as the availability of robust molecular phylogenies, allow patterns of metabolic scaling to be examined in evolutionary context.

We next identify three research areas predicated on the quantification of brain metabolism.

### Metabolic Tradeoffs

The expensive tissue hypothesis predicts a tradeoff in investment in the brain and other tissues (Aiello and Wheeler, 1995) and according to selfish brain theory, the brain exhibits resilience to metabolic stress by demanding energy from other systems (Peters et al., 2013). The immune system is also considered to be energetically costly and may exhibit energetic tradeoffs with the brain (Yamagata et al., 2017). These theories overlap conceptually, but have yet to be fully integrated. Insect brains may trade the cost of neural tissue with energetically costly flight muscles (Suarez, 2000; Schippers et al., 2010) or mandibular muscles (Roces and Lighten, 1995). Diversity in diet and sociality may affect systems tradeoffs: carbohydrate diets and predation are associated with alimentary tract adaptations for food exchange and alloparenting. Diet can affect energy availability, and cognitive needs may affect brain size and metabolism. Comparative studies will offer insights into energetic constraints on brain investment relative to nutritional ecology and sociality.

### Multilevel Scaling Analyses

Metabolic scaling should be considered for whole brains, functionally specialized brain compartments, workers, and colonies. Compartmental allometries can reflect variation in cognitive demands associated with division of labor (Muscedere and Traniello, 2012; Gordon et al., 2017, 2019), but the energy needs of brain compartments associated with task specialization are unknown. Hypometric metabolic scaling at the colony level has been demonstrated: workers in larger colonies with lower mass-specific metabolic rates may perform tasks that have lower costs (Shik, 2010; Fewell and Harrison, 2016; Waters et al., 2017). Data on how brain metabolism scales with colony size, combined with studies on colony size-related shifts in task distributions, can reveal the role of behavior in colony-level metabolic scaling.

### Genetic and Genomic Approaches to Metabolism

Neurons meet energy demands primarily through oxidative phosphorylation, and cytochrome oxidase (COX) has been used as a proxy for neuronal metabolism (Wong-Riley, 1989; Déglise et al., 2003). COX subunit isoforms may influence cellular metabolism (Taanman et al., 1994; Kadenbach, 2017; Chicherin et al., 2019, Reguera et al., 2020). Ants and other insects exhibit genetic diversity in COX (Liu and Beckenbach, 1992; Lunt et al., 1996) that may affect metabolic efficiency (Sabir et al., 2019).

## Conclusion

Despite the importance of brain metabolism, the relationship between brain metabolic scaling and sociality is not well understood. Hypometric scaling of metabolism to body size and colony size in ants may be due to energy efficiency associated with social complexity. However, the impact of social complexity on worker and colony-level metabolism is unclear. Additionally, synaptic plasticity is associated with age-related changes in brain size and behavioral repertoire, but associated metabolic costs are unknown. The striking variation in body size, brain size, and individual worker and collective behavior in ants can facilitate metabolic analysis at multiple levels to test theories of brain evolution. Metabolic variation—from genes and proteins to workers and colonies—can be assessed in relation to social organization.

## Author Contributions

ZC and JT wrote the manuscript. JT secured funding. Both authors contributed to the article and approved the submitted version.

## Conflict of Interest

The authors declare that the research was conducted in the absence of any commercial or financial relationships that could be construed as a potential conflict of interest.

## References

[B1] Aiello L. C. Wheeler P. (1995). The Expensive-tissue hypothesis: the brain and the digestive system in human and primate evolution. Curr. Anthro. 36, 199–221. 10.1086/204350

[B2] Arganda S. Hoadley A. P. Razdan E. S. Muratore I. M. Traniello J. F. A. (2020). The neuroplasticity of division of labor: worker polymorphism, compound eye structure and brain organization in the leafcutter ant Atta cephalotes. J. Comp. Physiol. A. 206, 651–662. 10.1007/s00359-020-01423-9 32506318

[B3] Arganda-Carreras I. Gordon D. G. Arganda S. Beaudoin M. Traniello J. F. A. (2017). Group-wise 3D registration based templates to study the evolution of ant worker neuroanatomy, in: *IEEE 14th International Symposium on Biomedical Imaging* (Melbourne, VIC).

[B4] Attwell D. Laughlin S. B. (2001). An energy budget for signaling in the grey matter of the brain. J. Cereb. Blood. Flow. Metab. 21, 1133–1145. 10.1097/00004647-200110000-00001 11598490

[B5] Barros L. F. Bolaños J. P. Bonvento G. Bouzier-Sore A.-K. Brown A. Hirrlinger J.. (2018). Current technical approaches to brain energy metabolism. Glia. 66, 1138–1159. 10.1002/glia.23248 29110344 PMC5903992

[B6] Cardona A. Saalfeld S. Preibich S. Schmid B. Cheng A. Pulokas J.. (2010). An integrated micro- and macroarchitectural analysis of the Drosophila brain by computer-assisted serial section electron microscopy. PLoS Biol. 8:e1000502. 10.1371/journal.pbio.1000502 20957184 PMC2950124

[B7] Chicherin I. V. Dashinimaev E. Baleva M. Krasheninnikov I. Levitskii S. Kamenski P. (2019). Cytochrome c oxidase on the crossroads of transcriptional regulation and bioenergetics. Front. Physiol. 10:644. 10.3389/fphys.2019.00644 31231235 PMC6558401

[B8] Darveau C. A. Suarez R. K. Andrews R. D. Hochachka P. W. (2002). Allometric cascade as a unifying principle of body mass effects on metabolism. Nature. 417, 166–170. 10.1038/417166a 12000958

[B9] DeCasien A. R. Higham J. P. (2019). Primate mosaic brain evolution reflects selection on sensory and cognitive specialization. Nat. Ecol. Evol. 3, 1483–1493. 10.1038/s41559-019-0969-0 31548648

[B10] Déglise P. Dacher M. Dion E. Gauthier M. Armengaud C. (2003). Regional brain variations of cytochrome oxidase staining during olfactory learning in the honeybee (*Apis mellifera*). *Behav. Neurosci*. 117, 540–547. 10.1037/0735-7044.117.3.540 12802882

[B11] Dunbar R. I. M. Shultz S. (2017). Why are there so many explanations for primate brain evolution? *Philos. Trans. R. Soc. Lond. B. Biol. Sci*. 372m 20160244. 10.1098/rstb.2016.0244 PMC5498304 28673920

[B12] Eberhard W. G. Wcislo W. T. (2011). Grade changes in brain-body allometry: morphological and behavioural correlates of brain size in miniature spiders, insects and other invertebrates. Adv. Insect. Physiol. 40, 155–214. 10.1016/B978-0-12-387668-3.00004-0

[B13] Faisal A. A. White J. A. Laughlin S. B. (2005). Ion-channel noise places limits on the miniaturization of the brain's wiring. Curr biol. 15, 1143–1149. 10.1016/j.cub.2005.05.056 15964281

[B14] Falibene A. Roces F. Rössler W. (2015). Long-term avoidance memory formation is associated with a transient increase in mushroom body synaptic complexes in leaf-cutting ants. Front. Behav. Neurosci. 9:84. 10.3389/fnbeh.2015.00084 25904854 PMC4389540

[B15] Feinerman O. Traniello J. F. A. (2015). Social complexity, diet, and brain evolution: modeling the effects of colony size, worker size, brain size, and foraging behavior on colony fitness in ants. Behav. Ecol. Sociobiol. 70, 1063–1074. 10.1007/s00265-015-2035-5

[B16] Fewell J. H. Harrison J. F. (2016). Scaling of work and energy use in social insect colonies. Behav. Ecol. Sociobiol. 70, 1047–1061. 10.1007/s00265-016-2097-z

[B17] Fonseca-Azevedo K. Herculano-Houzel S. (2012). Metabolic constraint imposes tradeoff between body size and number of brain neurons in human evolution. Proc. Natl. Acad. Sci. U. S. A. 109, 18571–18576. 10.1073/pnas.1206390109 23090991 PMC3494886

[B18] Giraldo Y. M. Patel E. Gronenberg W. Traniello J. F. A. (2013). Division of labor and structural plasticity in an extrinsic serotonergic mushroom body neuron in the ant Pheidole dentata. Neurosci. Lett. 534, 107–111. 10.1016/j.neulet.2012.11.057 23274482

[B19] Giurfa M. (2019). Honeybees foraging for numbers. J. Comp. Physiol. A. 205, 439–450. 10.1007/s00359-019-01344-2 31134327

[B20] Godfrey R. K. Gronenberg W. (2019). Brain evolution in social insects: advocating for the comparative approach. J. Comp. Physiol. A. 205, 13–32. 10.1007/s00359-019-01315-7 30656420

[B21] Gordon D. G. Ilieş J. Traniello J. F. A. (2017). Behavior, brain, and morphology in a complex insect society: trait integration and social evolution in the exceptionally polymorphic ant Pheidole rhea. Behav. Ecol. Sociobiol. 71, 166. 10.1007/s00265-017-2396-z

[B22] Gordon D. G. Traniello J. F. A. (2018). Synaptic organization and division of labor in the exceptionally polymorphic ant *Pheidole rhea*. Neurosci. Lett. 676, 46–50. 10.1016/j.neulet.2018.04.001 29625207

[B23] Gordon D. G. Zelaya A. Arganda-Carreras I. Arganda S. Traniello J. F. A. (2019). Division of labor and brain evolution in insect societies: Neurobiology of extreme specialization in the turtle ant Cephalotes varians. PLoS One 14:e0213618. 10.1371/journal.pone.0213618 30917163 PMC6436684

[B24] Gordon D. G. Zelaya A. Ronk K. Traniello J. F. A. (2018). Interspecific comparison of mushroom body synaptic complexes of dimorphic workers in the ant genus Pheidole. Neurosci. Lett. 662, 110–114. 10.1016/j.neulet.2017.10.009 29024727

[B25] Groh C. Kelber K. Grübel K. Rössler W. (2014). Density of mushroom body synaptic complexes limits intraspecies brain miniaturization in highly polymorphic leaf-cutting ant workers. Proc. Biol. Sci. 281:20140432. 10.1098/rspb.2014.0432 24807257 PMC4024300

[B26] Groh C. Rössler W. (2011). Comparison of microglomerular structures in the mushroom body calyx of neopteran insects. Arthropod. Struct. Dev. 40:358–367. 10.1016/j.asd.2010.12.002 21185946

[B27] Groh C. Rössler W. (2020). Analysis of synaptic microcircuits in the mushroom bodies of the honey bee. Insects 11, 43. 10.3390/insects11010043 PMC7023465 31936165

[B28] Groothius J Schmid H. M. (2017). Nasonia parasitic wasps escape from Haller's Rule by diphasic, partially isometric brain-body size scaling and selective neuropil adaptations. Brain. Behav. Evol. 90, 243–254. 10.1159/000480421 29059675 PMC5804836

[B29] Harris J. J. Jolivet R. Attwell D. (2012). Synaptic energy use and supply. Neuron. 75, 762–777. 10.1016/j.neuron.2012.08.019 22958818

[B30] Harrison J. (2017). Do performance-safety tradeoffs cause hypometric scaling in animals? Trends. Ecol. Evol. 32, 653–664. 10.1016/j.tree.2017.05.008 28760361

[B31] Harrison K. H. Hof P. R. Wang S. S.-H. (2002). Scaling laws in the mammalian neocortex: does form provide clues to function? J. Neurocytol. 31, 289–298. 10.1023/A:1024178127195 12815248

[B32] Healy S. D. Rowe C. (2007). A critique of comparative studies of brain size. Proc. Biol. Sci. 274, 453–464. 10.1098/rspb.2006.3748 17476764 PMC1766390

[B33] Hoops D. Vidal-Garcia M. Ullmann J. F. P. Janke A. L. Stait-Gardner T. Duchêne D. A.. (2017). Evidence for concerted and mosaic brain evolution in dragon lizards. Brain. Behav. Evol. 90, 211–223. 10.1159/000478738 28869944

[B34] Isler K. van Schaik C. P. (2006). Metabolic costs of brain size evolution. Biol. Lett. 2, 557–560. 10.1098/rsbl.2006.0538 17148287 PMC1834002

[B35] Isler K. van Schaik C. P. (2009). The expensive brain: a framework for explaining evolutionary changes in brain size. J. Hum. Evol. 57, 392–400. 10.1016/j.jhevol.2009.04.009 19732937

[B36] Kadenbach B. (2017). Regulation of mammalian 13-subunit cytochrome c oxidase and binding of other proteins: role of NDUFA4. Trends Endocrinol. Metab. 28, 761–770. 10.1016/j.tem.2017.09.003 28988874

[B37] Kamhi J. F. Gronenberg W. Robson S. K. A. Traniello J. F. A. (2016). Social complexity influences brain investment and neural operation costs in ants. Proc. Biol. Sci. 283, 20161949. 10.1098/rspb.2016.1949 27798312 PMC5095391

[B38] Kiesow H. Dunbar R. I. M. Kable J. W. Kalenscher T. Vogeley K. Schilbach L.. (2020). 10,000 social brains: Sex differentiation in human brain anatomy. Sci. Adv. 6:eaaz1170. 10.1126/sciadv.aaz1170 32206722 PMC7080454

[B39] Kolb E. M. Rezende E. L. Holness L. Radtke A. Lee S. K. Obenaus A.. (2013). Mice selectively bred for high voluntary wheel running have larger mid-brains: support for the mosaic model of brain evolution. J. Exp. Biol. 216, 515–523. 10.1242/jeb.076000 23325861

[B40] Kotrschal A. Lievens E. J. P. Dahlbom J. Bundsen A. Semenova S. Sundvik M.. (2014). Artificial selection on relative brain size reveals a positive genetic correlation between brain size and proactive personality in the guppy. Evolution 68, 1139–1149. 10.1111/evo.12341 24359469 PMC4285157

[B41] Laughlin S. B. de Ruyter van Steveninck R. R. Anderson J. C. (1998). The metabolic cost of neural information. Nat neurosci. 1, 36–41. 10.1038/236 10195106

[B42] Liao W. B. Lou S. L. Zeng Y. Merilä J. (2015). Evolution of anuran brains: disentangling ecological and phylogenetic sources of variation. J. Evol. Biol. 28, 1986–1996. 10.1111/jeb.12714 26248891

[B43] Lighton J. R. B. (2019). Measuring Metabolic Rates: A Manual for Scientists. New York, NY: Oxford University Press.

[B44] Liu H. Beckenbach A. T. (1992). Evolution of the mitochondrial cytochrome oxidase II gene among 10 orders of insects. Mol. Phylogenet. Evol. 1, 41–52. 10.1016/1055-7903(92)90034-E 1342923

[B45] Logan C. J. Avin S. Boogert N. Buskell A. Cross F. R. Currie A.. (2018). Beyond brain size: uncovering the neural correlates of behavioral and cognitive specialization. Comp. Cogn. Behav. Rev. 13, 55–89. 10.3819/CCBR.2018.130008

[B46] Lunt D. H. Zhang D.-X. Szymura J. M. Hewitt G. M. (1996). The insect cytochrome oxidase I gene: evolutionary patterns and conserved primers for phylogenetic studies. Insect Mol. Biol. 5, 153–165. 10.1111/j.1365-2583.1996.tb00049.x 8799733

[B47] Mertl A. L. Traniello J. F. A. (2009). Behavioral evolution in the major worker subcaste of twig-nesting Pheidole (Hymenoptera: Formicidae): does morphological specialization influence task plasticity? Behav. Ecol. Sociobiol. 63, 1411–1426. 10.1007/s00265-009-0797-3

[B48] Muscedere M. L. Johnson N. Gillis B. C. Kamhi J. F. Traniello J. F. A. (2012). Serotonin modulates worker responsiveness to trail pheromone in the ant *Pheidole dentata*. J. Comp. Physiol. A. 198, 219–227. 10.1007/s00359-011-0701-2 22134381

[B49] Muscedere M. L. Traniello J. F. A. (2012). Division of labor in the hyperdiverse ant genus Pheidole is associated with distinct subcaste- and age-related patterns of worker brain organization. PLoS One 7:e31618. 10.1371/journal.pone.0031618 22363686 PMC3281964

[B50] Muscedere M. L. Willey T. A. Traniello J. F. A. (2009). Age and task efficiency in the ant Pheidole dentata: young minor workers are not specialist nurses. Anim. Behav. 77, 911–918. 10.1016/j.anbehav.2008.12.018

[B51] Navarrete A. van Schaik C. P. Isler K. (2011). Energetics and the evolution of human brain size. Nature. 480, 91–93. 10.1038/nature10629 22080949

[B52] Neville K. E. Bosse T. L. Klekos M. Mills J. F. Weicksel S. E. Waters J. S.. (2017). A novel ex vivo method for measuring whole brain metabolism in model systems. J. Neurosci. Methods 296, 32–43. 10.1016/j.jneumeth.2017.12.020 29287743 PMC5840572

[B53] New A. S. Buchsbaum M. S. Hazlett E. A. Goodman M. Koenigsberg H. W. Lo J.. (2004). Fluoxetine increases relative metabolic rate in prefontal cortex in impulsive aggression. Psychopharmacology (Berl) 176, 451–458. 10.1007/s00213-004-1913-8 15160265

[B54] Niven J. E. Farris S. M. (2012). Miniaturization of nervous systems and neurons. Curr Biol. 22, R323–9. 10.1016/j.cub.2012.04.002 22575474

[B55] Niven J. E. Laughlin S. B. (2008). Energy limitation as a selective pressure on the evolution of sensory systems. J. Exp. Biol. 211, 1792–1804. 10.1242/jeb.017574 18490395

[B56] Noonan M. P. Mars R. B. Sallet J. Dunbar R. I. M. Fellows L. K. (2018). The structural and functional brain networks that support human social networks. Behav. Brain. Res. 355, 12–23. 10.1016/j.bbr.2018.02.019 29471028 PMC6152579

[B57] Ott S. R. Rogers S. M. (2010). Gregarious desert locusts have substantially larger brains with altered proportions compared with the solitarious phase. Proc. Biol. Sci. 277, 3087–3096. 10.1098/rspb.2010.0694 20507896 PMC2982065

[B58] Peters A. Kubera B. Hubold C. Langemann D. (2013). The corpulent phenotype - how the brain maximizes survival in stressful environments. Front. Neurosci. 7:47. 10.3389/fnins.2013.00047 23565074 PMC3613700

[B59] Polilov A. A. Makarova A. A. (2017). The scaling and allometry of organ size associated with miniaturization in insects: a case study for Coleoptera and Hymenoptera. Sci. Rep. 22, 43095. 10.1038/srep43095 28225037 PMC5320524

[B60] Pontzer H. Brown M. H. Raichlen D. A. Dunsworth H. Hare B. Walker K.. (2016). Metabolic acceleration and the evolution of human brain size and life history. Nature 533, 390–392. 10.1038/nature17654 27144364 PMC4942851

[B61] Prothero J. W. (2015). The Design of Mammals: A Scaling Approach. Cambridge: Cambridge University Press.

[B62] Reguera D. P. Cunátov,á K. Vrback,ý M. Pecinov,á A. Houštěk J. Mráček T.. (2020). Cytochrome c oxidase subunit 4 isoform exchange results in modulation of oxygen affinity. Cells 9, 443. 10.3390/cells9020443 32075102 PMC7072730

[B63] Rittschof C. C. Grozinger C. M. Robinson G. E. (2015). The energetic basis of behavior: bridging behavioral ecology and neuroscience. Curr. Opin. Behav. Sci. 6, 19–37. 10.1016/j.cobeha.2015.07.006

[B64] Rittschof C. C. Schirmeier S. (2017). Insect models of central nervous system metabolism and its links to behavior. Glia 66, 1–16. 10.1002/glia.23235 28960551

[B65] Rittschof C. C. Vekaria H. J. Palmer J. H. Sullivan P. G. (2019). Biogenic amines and activity levels alter the neural energetic response to aggressive social cues in the honey bee *Apis mellifera*. J. Neurosci. Res. 97, 991–1003. 10.1002/jnr.24443 31090236

[B66] Riveros A. J. Seid M. A. Wcislo W. T. (2012). Evolution of brain size in class-based societies of fungus growing ants (Attini). Anim. Behav. 83, 1043–1049. 10.1016/j.anbehav.2012.01.032

[B67] Roces F. Lighten J. R. B. (1995). Larger bites of leaf-cutting ants. Nature 373, 392. 10.1038/373392a0

[B68] Rössler W. (2019). Neuroplasticity in desert ants (Hymenoptera: Formicidae) - importance for the ontogeny of navigation. *Myrmecol*. News 29, 1–20. 10.25849/myrmecol.news_029:001

[B69] Sabir J. S. M. Rabah S. Yacoub H. Hajrah N. H. Atef A. Al-Matary M.. (2019). Molecular evolution of cytochrome c oxidase-i protein of insects living in Saudi Arabia. PLoS One 14:e0224336. 10.1371/journal.pone.0224336 31682609 PMC6827904

[B70] Sasaki T. Pratt S. C. (2018). The Psychology of Superorganisms: Collective decision making by insect societies. Annu. Rev. Entomol. 63, 259–275. 10.1146/annurev-ento-020117-043249 28977775

[B71] Schippers M.-P. Dukas R. McClelland G. B. (2010). Lifetime and caste specific changes in flight metabolic rate and muscle biochemistry of honeybees, *Apis mellifera*. J. Comp. Physiol. B 180, 45–55. 10.1007/s00360-009-0386-9 19578858

[B72] Seid M. A. Castillo A. Wcislo W. T. (2011). The allometry of brain miniaturization in ants. Brain. Behav. Evol. 77, 5–13. 10.1159/000322530 21252471

[B73] Seid M. A. Goode K. Li C. Traniello J. F. A. (2008). Age- and subcaste- related patterns of serotonergic immunoreactivity in the optic lobes of Pheidole dentata. Dev. Neurobiol. 68, 1325–1333. 10.1002/dneu.20663 18666203

[B74] Seid M. A. Harris K. M. Traniello J. F. A. (2005). Age-related changes in the number and structure of synapses in the lip region of the mushroom bodies in the ant *Pheidole dentata*. J. Comp. Neurol. 488, 269–277. 10.1002/cne.20545 15952165

[B75] Seid M. A. Traniello J. F. A. (2005). Age-related changes in biogenic amines in individual brains of the ant Pheidole dentata. Naturwissenschaften 92, 198–201. 10.1007/s00114-005-0610-8 15776257

[B76] Seid M. A. Traniello J. F. A. (2006). Age-related repertoire expansion and division of labor in Pheidole dentata (Hymenoptera: Formicidae): a new perspective on temporal polyethism and behavioral plasticity in ants. Behav. Ecol. Sociobiol. 60, 631–644. 10.1007/s00265-006-0207-z

[B77] Sengupta B. Stemmler M. B. Friston K. J. (2013). Information and efficiency in the nervous system - a synthesis. PLoS Comput Biol. 9:e1003157. 10.1371/journal.pcbi.1003157 23935475 PMC3723496

[B78] Shik J. Z. (2010). The metabolic costs of building ant colonies from variably sized subunits. Behav. Ecol. Sociobiol. 64, 1981–1990. 10.1007/s00265-010-1009-x

[B79] Suarez R. K. (2000). Energy metabolism during insect flight: biochemical design and physiological performance. Physol. Biochem. Zool. 73, 765–771. 10.1086/318112 11121349

[B80] Taanman J. W. Turina P. Capaldi R. A. (1994). Regulation of cytochrome c oxidase by interaction of ATP at two binding sites, one on subunit VIa. Biochemistry 33, 11833–11841. 10.1021/bi00205a020 7918401

[B81] Todorova V. Blokland A. (2017). Mitochondria and synaptic plasticity in the mature and aging nervous system. Curr. Neuropharmacol. 15, 166–173. 10.2174/1570159X14666160414111821 27075203 PMC5327446

[B82] van der Woude E. Smid H. M. Chittka L. Huigens M. E. (2013). Breaking Haller's Rule: brain-body size isometry in a minute parasitic wasp. Brain. Behav. Evol. 81, 86–92. 10.1159/000345945 23363733

[B83] Waters J. S. Ochs A. Fewell J. H. Harrison J. F. (2017). Differentiating causality and correlation in allometric scaling: ant colony size drives metabolic hypometry. Proc. Biol. Sci. 284, 20162582. 10.1098/rspb.2016.2582 28228514 PMC5326530

[B84] Wehner R. Fukushi T. Isler K. (2007). On being small: brain allometry in ants. Brain. Behav. Evol. 69, 220–228. 10.1159/000097057 17108673

[B85] West G. B. Brown J. H. (2005). The origin of allometric scaling laws in biology from genomes to ecosystems: towards a quantitative unifying theory of biological structure and organization. J. Exp. Biol. 208, 1575–1592. 10.1242/jeb.01589 15855389

[B86] West G. B. Woodruff W. H. Brown J. H. (2002). Allometric scaling of metabolic rate from molecules and mitochondria to cells and mammals. Proc. Natl. Acad. Sci. U. S. A. 99, 2473–2478. 10.1073/pnas.012579799 11875197 PMC128563

[B87] Wong-Riley M. T. (1989). Cytochrome oxidase: an endogenous metabolic marker for neuronal activity. Trends. Neurosci. 12, 94–101. 10.1016/0166-2236(89)90165-3 2469224

[B88] Yamagata A. S. Mansur R. B. Rizzo L. B. Rosenstock T. McIntyre R. S. Brietzke E. (2017). Selfish brain and selfish immune system interplay: a theoretical framework for metabolic comorbidities of mood disorders. Neurosci. Biobehav. Rev. 72, 43–49. 10.1016/j.neubiorev.2016.11.010 27871787

[B89] Yilmaz A. Lindenberg A. Albert S. Grübel K. Spaethe J. Rössler W.. (2016). Age-related and light-induced plasticity in opsin gene expression and in primary and secondary visual centers of the nectar-feeding ant *Camponotus rufipes*. Dev. Neurobiol. 76, 1041–1057. 10.1002/dneu.22374 26724470

